# Potential Mechanisms Underlying the Minimal Impact of Cry1Ab1 Protein on *Myzus persicae*

**DOI:** 10.3390/ijms26072924

**Published:** 2025-03-24

**Authors:** Liang Jin, Binwu Zhang, Luis Carlos Ramos Aguila, Jingwen Lu, Xueke Gao, Junyu Luo, Jinjie Cui, Yi Lin

**Affiliations:** 1 Research Base of Zhengzhou University, State Key Laboratory of Cotton Bio-Breeding and Integrated Utilization, Institute of Cotton Research, Chinese Academy of Agricultural Sciences, Anyang 455000, China; 2 Fujian Provincial Key Laboratory of Biochemical Technology, Department of Bioengineering & Biotechnology, College of Chemical Engineering, Huaqiao University, Xiamen 361021, China; 3 State Key Laboratory of Ecological Pest Control for Fujian and Taiwan Crops, College of Plant Protection, Fujian Agriculture and Forestry University, Fuzhou 350002, China

**Keywords:** *Bacillus thuringiensis*, Cry1Ab1 protein, Cry-binding proteins, *Myzus persicae*, non-target

## Abstract

Transgenic crops have been commercially cultivated for nearly three decades, leading to increasing concerns about their environmental safety, particularly their effects on non-target organisms. This study investigated the underlying mechanisms behind the lack of impact of the Cry1Ab1 protein on the *Myzus persicae*. The Cry1Ab1 protein showed no significant impact on the survival and development of *M. persicae*. Compared to other Cry protein, fewer Cry1Ab1-binding proteins were identified including beta-actin, ATP synthase subunit alpha, and GPN-loop GTPase 2. Transcriptomic analysis showed that a small set of pathways, mainly involved in immune defense, were temporarily enriched at 24 h after exposure to the Cry1Ab1 protein, while no significant pathways were enriched at 48 h in *M. persicae*. The results suggest that the Cry1Ab1 protein has a transient and minimal impact on *M. persicae*. Further structural comparisons between Cry1Ab1 and other Cry proteins (e.g., Cry1Ac) revealed significant differences in Domain III, which likely reduced the binding efficiency and impact on *M. persicae*’s metabolism and biological traits. This study provides valuable insights into the molecular and functional mechanisms behind the ineffectiveness of Cry1Ab1 on *M. persicae* and contributes to the safety evaluation of Bt for non-target organisms.

## 1. Introduction

The cultivation area of genetically modified (GM) crops has increased by 121-fold from 1996 to 2023, now accounting for approximately 13.38% of global farmland (1542 million hectares). The total area planted with GM crops has now surpassed 3.4 billion hectares [1]. Particularly, transgenic crops containing *Bacillus thuringiensis* (Bt) genes, such as soybeans, cotton, and corn are the primary crops planted [1]. In recent years, the extensive use of Bt pesticides has continued to rise, and their proportion in China’s microbial pesticide market has exceeded 95% [2]. Bt, a type of gram-positive bacteria, exhibits a wide global distribution [3]. During the spore formation process, Bt produces parasporal crystals, primary composed of insecticidal crystal proteins such as Cyt and Cry proteins [4]. The insecticidal mechanisms of the most studied Cry proteins have gradually been elucidated. A key step in this process is the interaction of these proteins with specific receptors on the midgut brush border membrane vesicles (BBMVs) of insects, such as cadherin (CAD), aminopeptidase N (APN), alkaline phosphatase (ALP), and ABC transporter [2,5]. In recent years, certain progress has been made in understanding Cry protein binding in Lepidoptera. For instance, HaATPs-α has been identified as a potential receptor for Cry1Ac in *Helicoverpa armigera* [6], while ABCC2 has been recognized as a functional receptor for Cry1Ca in *Spodoptera litura* [7]. Functional screening of Cry protein domains revealed that Domain I is essential for the binding of Cry2Aa to the midgut of *H. armigera* [8]. Additionally, Cry-binding proteins or receptor-binding proteins present in organisms may interfere with the action of Cry proteins. For instance, Cry6Aa was found to bind to aspartic protease-1 (ASP-1) in *Caenorhabditis elegans*, resulting in necrotic cell death that is dependent on ASP-1 [9]. Zhao et al. [10] revealed that Cry41-related protein interacts with Cathepsin B and enhances its activity, causing acceleration of apoptosis of aphid cells. Galectin-14 has been reported to compete with Cry11Aa for binding to BBMVs and ALP1 in *Aedes aegypti*, thereby preventing the effective binding of Cry protein to its receptors and ultimately altering the toxicity of Cry11Aa [11]. Therefore, differences in associated binding proteins can modify the behavior of Cry proteins, leading to varied impacts.

Aphids are a type of sucking insect belonging to the order Hemiptera. Approximately 4700 aphid species have been identified, with around 450 known to cause damage to crops [12]. Aphids use their sharp mouthparts to suck plant sap, and the chlorophyllase and pectinase enzymes in their saliva can adversely affect plant growth and development [13]. Furthermore, aphids are key vectors for plant viruses [14]. For instance, *Myzus persicae* has been shown to transmit over 100 different plant viruses [15]. Despite the specific insecticidal activity of Cry proteins against various agricultural and forestry pests, most of these proteins are ineffective against aphids [16]. With the large-scale planting of Bt transgenic crops, environmental safety assessments have become a major focus [17]. The effects of Bt Cry proteins on non-target aphids are an active area of research [18,19,20]. Some studies have reported that certain Cry proteins impact the fecundity and development of aphids [21,22,23], while other Cry proteins like Cry1Ab have no significant effect on various aphids [18,24,25]. However, the underlying reasons for these differences have not been systematically reported.

This study aimed to investigate the potential factors affecting aphid development in response to Cry protein treatment, using the typical Cry1Ab1 (Cry1Ab) protein to examine its effects on the highly destructive aphid *M. persicae*. Initially, a bioassay was conducted to assess the toxicity of the Cry1Ab1 protein against *M. persicae*. Subsequently, proteins bound with Cry1Ab1 were identified using pull-down and Liquid Chromatography-Tandem Mass Spectrometry (LC-MS/MS) assays. These identified proteins were then analyzed for their associated functions using the STRING database. Transcriptome sequencing was performed to reveal the responses of *M. persicae* to the Cry1Ab1 protein. Furthermore, sequence and structural comparisons of the Cry1A class proteins Cry1Ab1 and Cry1Ac revealed significant differences in Domain Ⅲ. These differences might underlie the differential effects observed in *M. persicae*.

## 2. Results

### 2.1. Cry1Ab1 Protein Rarely Kills M. persicae

**Figure 1:**
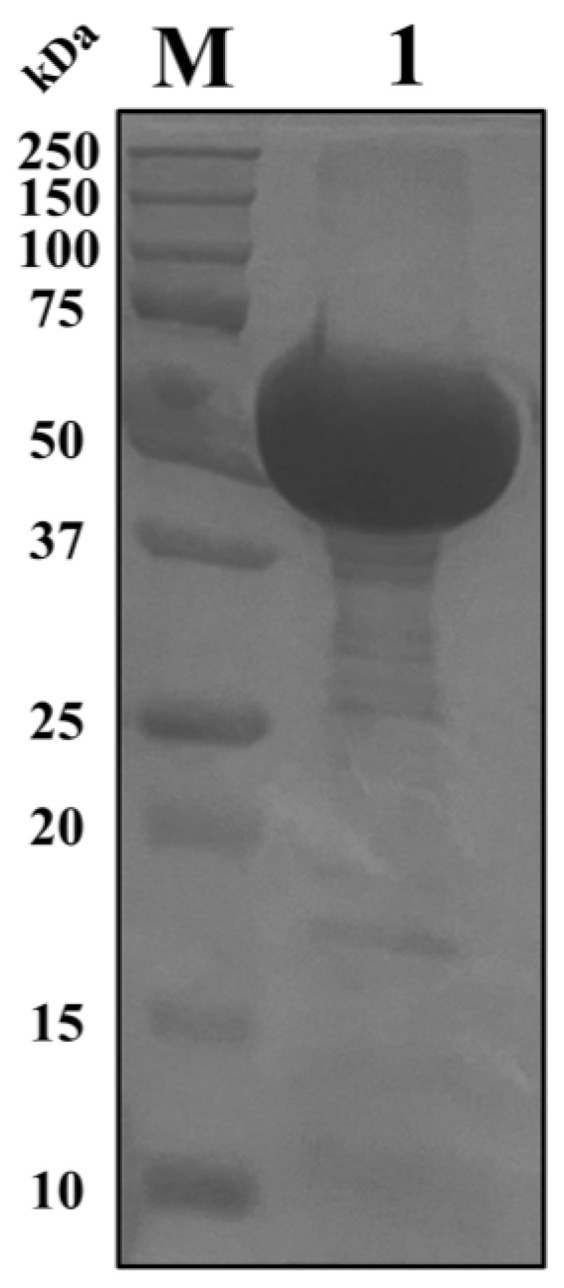
SDS–PAGE analysis of Cry1Ab1 protein. M, Protein Marker; 1, Purified Cry1Ab1 protein.

**Figure 2:**
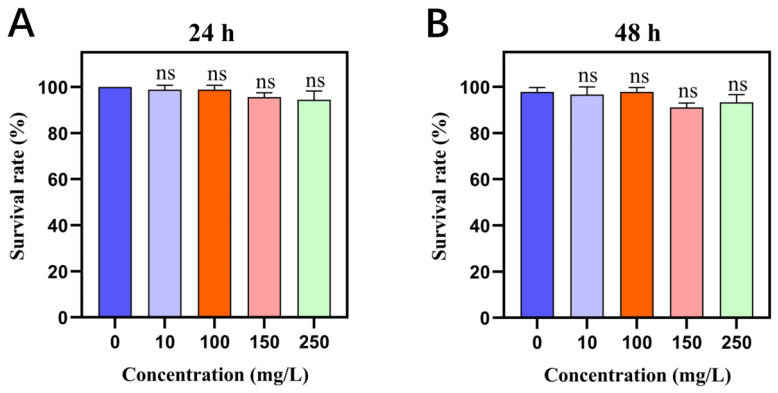
Toxicity assessment of Cry1Ab1 protein on *M. persicae.* Fourth instar *M. persicae* nymphs were exposed to increasing concentrations of Cry1Ab1 protein (10, 100, 150, and 250 mg/L). After 24 h (**A**) and 48 h (**B**), survival rates were recorded and analyzed using GraphPad Prism 8. Statistical significance between the control and treated groups was assessed via one-way ANOVA with Dunnett’s post hoc test, revealing no significant differences (ns).

As illustrated in Figure 1, the purified Cry1Ab1 protein was obtained following the expression, purification, and concentration steps. Subsequently, bioassays against *M. persicae* were conducted using four different concentrations of the purified Cry1Ab1 protein, employing both the leaf-dip method (Figure 2) and the membrane capsule method (Figure S1). The survival rates of *M. persicae* exposed to various concentrations of Cry1Ab1 protein did not differ significantly from those of the control group, indicating that the Cry1Ab1 protein had minimal lethal effects on *M. persicae*. This finding was consistent with previously reported results [26,27].

### 2.2. Identification of Cry1Ab1-Binding Proteins

**Figure 3:**
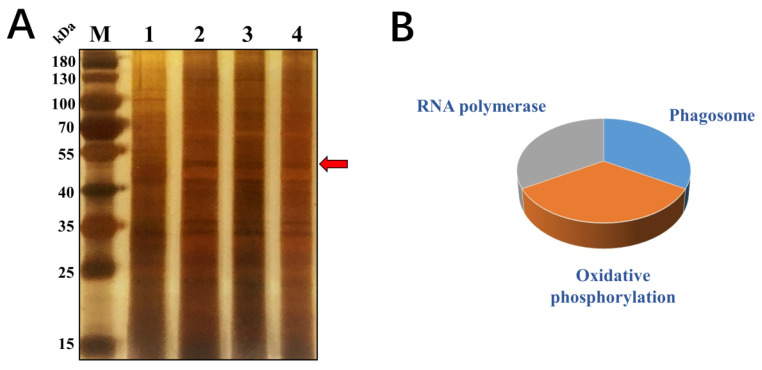
Isolation of Cry1Ab1-binding proteins in *M. persicae*. (**A**): M, Protein Marker; 1, Control; 2–4, Cry1Ab1-binding proteins (Results from the first to third pull-down). The red arrow indicates protein bands of Cry1Ab1-binding proteins including Beta-actin, GPN-loop GTPase 2, ATP synthase subunit alpha, and an uncharacterized protein. (**B**): The proteins bound with Cry1Ab1 are mainly involved in RNA polymerase, phagocytosis, and oxidative phosphorylation.

Identifying Cry-binding proteins is essential for understanding the mechanisms of Cry protein action in insects [28,29]. These proteins that bound to the Cry1Ab1 protein were isolated using a pull-down assay, separated on SDS-PAGE gels, and subjected to silver staining (Figure 3A). The treatment group exhibited a distinct band at approximately 55 kDa when compared to the control group. Table 1 showed that the Cry1Ab1-binding proteins included beta-actin (β-actin), ATP synthase subunit alpha, GPN-loop GTPase 2 (Gpn2) from the GTPase protein family, and an unannotated protein. Interestingly, some of these proteins or their homologues have been previously identified as Cry-binding proteins. For example, V-ATPase subunit A and actin were identified as Cry1Ac-binding proteins in the midgut of *Heliothis virescens* [30]. V-ATPase subunit B and actin have been identified as Cry1Ac-binding proteins in *H. armigera* [31]. Therefore, ATPase and actin are likely to be common Cry-binding proteins in insects. These results also suggested that the protocol used in the present study was suitable for isolating and identifying Cry-binding proteins. Notably, Gpn2, a member of the GPN family of GTPases, was first described as a Cry-binding protein in this study. GTPases bind and hydrolyze GTP to produce GDP and inorganic phosphate, and they are involved in various cellular functions, including protein synthesis, cell differentiation, energy metabolism, and signal transduction [32,33,34].

Furthermore, the sequences of the proteins that bound to the Cry1Ab1 protein were submitted to the STRING server for pathway analysis. As shown in Figure 3B, the Cry1Ab1-binding proteins were primarily involved in phagocytosis, RNA polymerase, and oxidative phosphorylation, among others.

### 2.3. Transcriptome Sequencing and Assembly

Fourth instar *M. persicae* nymphs exposed to the Cry1Ab1 protein for 24 and 48 h as well as nymphs that received no protein treatment were collected for transcriptome sequencing (Figure S2). After filtering out noise and removing adapter sequences from the raw data, the clean data for each sample exceeded 6.37 Gb, with a Q30 base percentage of over 95.91% in each sample. The GC content ranged from 40.66% to 47.19%. Clean reads from each sample were mapped to reference sequences assembled using Trinity, and the mapping results were analyzed. The alignment rates across samples ranged from 91.31% to 94.64% (Table S1), indicating a high-quality assembly.

**Figure 4:**
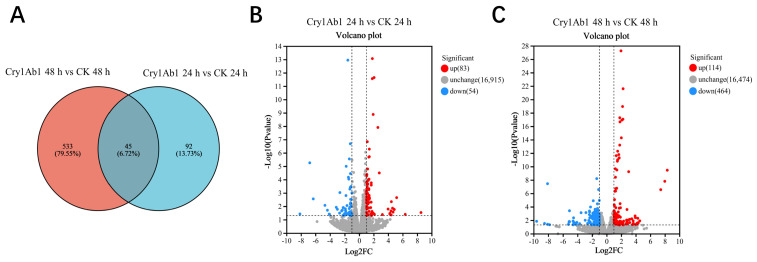
Effects of Cry1Ab1 treatment on gene expression of *M. persicae.* (**A**): Venn diagram displaying the number of DEGs in *M. persicae*; (**B**): Volcano plot illustrating DEGs in *M. persicae* larvae after 24 h of exposure to Cry1Ab1; (**C**): Volcano plot illustrating DEGs in *M. persicae* larvae after 48 h of exposure to Cry1Ab1. Red dots indicate significantly up-regulated genes, and blue dots indicate significantly down-regulated genes in the volcano plot.

The analysis of the differentially expressed genes (DEGs)was visualized using Venn diagrams and volcano plots (Figure 4). A total of 670 DEGs were identified between the Cry1Ab1-treated group and the control group. After 24 h of exposure to the Cry1Ab1 protein, 137 DEGs were detected, including 83 up-regulated and 54 down-regulated genes. Meanwhile, after 48 h of exposure to the Cry1Ab1 protein, 578 DEGs were identified, with 114 up-regulated and 464 down-regulated genes.

### 2.4. Functional Analysis of DEGs

**Figure 5:**
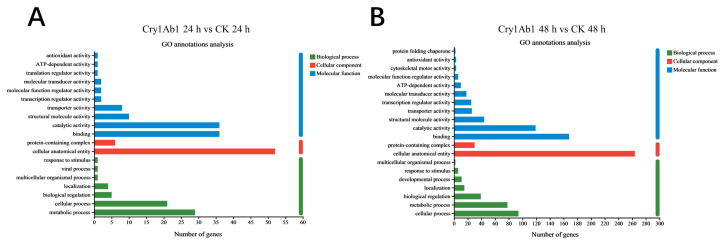
GO functional classification of DEGs in *M. persicae* after exposure to Cry1Ab1 protein. (**A**): GO functional classification of DEGs in *M. persicae* after 24 h of exposure to Cry1Ab1; (**B**): GO functional classification of DEGs in *M. persicae* after 48 h of exposure to Cry1Ab1.

To elucidate the genetic functions influenced by Cry1Ab1 protein exposure, the DEGs were categorized according to Gene Ontology (GO) annotations. The DEGs were classified into three primary categories: biological processes (BP), cellular components (CC), and molecular functions (MF). In the BP category, the DEGs predominantly impacted cellular processes, metabolic processes, biological regulation, and localization. In the CC category, the DEGs were mainly concentrated in cellular anatomical entities and protein-containing complexes. In the MF category, the DEGs were chiefly associated with binding, catalytic activity, structural molecule activity, transporter activity, transcription regulator activity, and molecular transducer activity (Figure 5).

**Figure 6:**
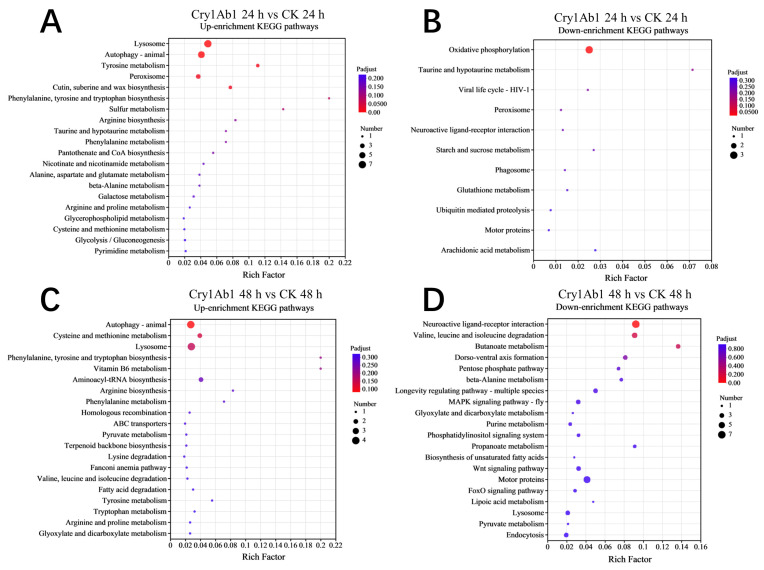
KEGG enrichment analysis of DEGs between Cry1Ab1-treated and control group. (**A**): the top up-enrichment KEGG pathways in the Cry1Ab1-treated group versus control group after 24 h of exposure; (**B**): the top down-enrichment KEGG pathways in the Cry1Ab1-treated group versus control group after 24 h of exposure; (**C**): the top up-enrichment KEGG pathways in the Cry1Ab1-treated group versus control group after 48 h of exposure; (**D**): the top down-enrichment KEGG pathways in the Cry1Ab1-treated group versus control group after 48 h of exposure.

Moreover, metabolic pathway analysis of the DEGs using the KEGG database could provide deeper insights into the impact of the Cry1Ab1 protein on *M. persicae* (Figure 6). In detail, various signaling pathways, including lysosome, autophagy—animal, tyrosine metabolism, peroxisome, and cutin, suberine, and wax biosynthesis, were significantly up-regulated (Figure 6A), while the oxidative phosphorylation pathway was significantly down-regulated (Figure 6B) compared to the control group after 24 h of Cry1Ab1 exposure. Additionally, after 48 h of Cry1Ab1 exposure, many signaling pathways, including autophagy—animal, cysteine and methionine metabolism, and lysosome, were up-regulated, but without significant differences compared to the control group (Figure 6C). Also, neuroactive ligand-receptor interaction, valine, leucine, and isoleucine degradation, and butanoate metabolism were down-regulated, but without significant differences (Figure 6D).

### 2.5. Cluster Analysis of DEGs

**Figure 7:**
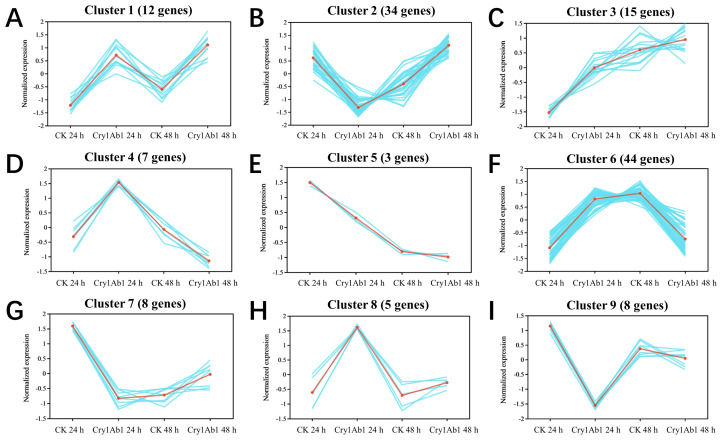
Cluster analysis patterns of DEGs. These DEGs were divided into nine distinct clusters based on the gene expression temporal patterns. Specifically, Cluster 1 comprises 12 DEGs (**A**), Cluster 2 contains 34 (**B**), Cluster 3 includes 15 (**C**), Cluster 4 has 7 (**D**), Cluster 5 consists of 3 (**E**), Cluster 6 encompasses 44 (**F**), Cluster 7 holds 8 (**G**), Cluster 8 involves 5 (**H**), and Cluster 9 contains 8 (**I**). The red line represents the overall expression trend of genes within each cluster, summarizing the expression dynamics of the cluster. The blue lines depict the specific expression trajectories of individual genes within each cluster.

All of the DEGs were classified into nine distinct co-expression pattern clusters (Figure 7). Cluster 6 had the highest number of DEGs (44), followed by Cluster 2 (34), Cluster 3 (15), Cluster 1 (12), Cluster 7 (8), Cluster 9 (8), Cluster 4 (7), Cluster 8 (5), and Cluster 5 (3). The DEGs in Cluster 1 were primarily enriched in tyrosine metabolism and significantly enriched in the lysosomal and autophagy processes in animals. Cluster 2 DEGs were mainly enriched in oxidative phosphorylation, peroxisome function, the HIV-1 viral life cycle, ubiquitin-mediated proteolysis, and starch and sucrose metabolism. Cluster 3 DEGs were involved in the largest number of pathways, with significant enrichment in arginine biosynthesis, cysteine and methionine metabolism, and arginine and proline metabolism. Pathways such as pyrimidine metabolism, nucleotide metabolism, and purine metabolism were also enriched, but no significant differences were observed. Cluster 4 DEGs were enriched in lysosomal processes, while Cluster 5 DEGs were enriched in neuroactive ligand-receptor interactions. Cluster 6 DEGs, which were enriched in the second largest number of pathways, showed significant enrichment in lysosomal processes, autophagy in animals, pantothenate and CoA biosynthesis, beta-alanine metabolism, taurine and hypotaurine metabolism, longevity-regulating pathways in multiple species, and peroxisome function. However, the phosphatidylinositol signaling system and sulfur metabolism did not show significant enrichment. Cluster 7 DEGs were predominantly enriched in arachidonic acid metabolism, glutathione metabolism, taurine and hypotaurine metabolism, motor protein function, and phagosome activity. Cluster 8 DEGs were mainly enriched in peroxisome function, cutin and suberin biosynthesis, glycerophospholipid metabolism, and insect hormone biosynthesis. In contrast, Cluster 9 DEGs were not enriched in any KEGG pathways.

## 3. Discussion

Transgenic crops have been commercially cultivated for nearly 30 years, leading to growing concerns about their environmental safety [17]. Their safety evaluation for non-target organisms has increasingly become a key area of research. Some Cry proteins, such as Cry4Aa, Cry11Aa, CryAc, and Cry1F, have been shown to significantly affect the development or fecundity of aphids [22,23], but worldwide use bio-insecticides Cry1Ab was reported to exhibit no effect on various aphids [18,20,27,35]. This study focused on exploring the potential reasons why Cry1Ab1 did not affect the notorious *M. persicae*. Different methods confirmed that the Cry1Ab1 protein was inactive against *M. persicae*. β-actin, ATP synthase subunit alpha, and Gpn2 were identified as the Cry1Ab1 binding proteins, and their associated functions including cell phagocytosis, RNA polymerase, cellular oxidative phosphorylation were also clarified in *M. persicae*. Through transcriptomic analysis, we further found that lysosome, autophagy—animal, tyrosine metabolism, peroxisome, and cutin, suberine and wax biosynthesis were significantly up-regulated. Oxidative phosphorylation pathways were significantly down-regulated at 24 h. There were no pathways of significantly different enrichment levels after 48 h of exposure to the Cry1Ab1 protein in *M. persicae*.

Lysosomes play a crucial role in the development of organisms, cell growth, differentiation, and defense against foreign substances [36]. Autophagy refers to the physiological process in which cells direct their components to the lysosome for degradation through autophagosomes [37]. Autophagy, recognized for maintaining cellular equilibrium through the degradation of proteins and organelles, is considered a crucial defense and stress response mechanism [38]. Studies indicated that autophagy and endocytosis pathways were linked to Bt proteins [39,40]. Intriguingly, Cry1Ab1-binding proteins are involved in cell phagocytosis. Phagocytosis is a highly conserved innate immune mechanism that not only eliminates foreign microbial pathogens but also digests apoptotic or necrotic cell debris from many cells. When phagocytosis occurs, foreign substances are recognized by cells, bound to the cell surface, and engulfed into the cells by phagosomes [41,42]. Hence, it was highly likely that the Cry1Ab1 protein induces an immune defense response such as autophagy in *M. persicae*, primarily through cell phagocytosis. Tyrosine metabolism is closely associated with insect cuticle hardening and innate immune responses [43]. Therefore, the up-regulation of lysosomes, autophagy—animal, and tyrosine metabolism suggests that the defense mechanism in *M. persicae* was likely activated after 24 h of exposure to the Cry1Ab1 protein. Peroxisomes, widely present in cellular organelles, play a vital role in numerous essential metabolic processes. They are acknowledged as key elements in aging, longevity, and age-related conditions [44]. Cutin, suberine, and wax biosynthesis was likely associated with the production of related substances and may regulate individual growth [45]. The up-regulation of peroxisome and cutin, suberine, and wax biosynthesis might cause transient changes in related substances within the *M. persicae*, because no metabolic pathways were significantly enriched after being exposed to Cry1Ab1 for 48 h. In addition, oxidative phosphorylation was simultaneously enriched in the functional pathways of the Cry1Ab1-binding proteins and the KEGG metabolic pathway of the DEGs. Oxidative phosphorylation refers to the process of releasing energy as a result of the oxidation of organic matter in the organism and drives the synthesis of ATP, which is necessary for sustaining the synthesis of chemicals that maintain metabolism and cell structure and for the transport of the ions and molecules that maintain the intracellular environment [46]. Thus, it was likely that the binding of Cry1Ab1 to the proteins related to oxidative phosphorylation down-regulated the oxidative phosphorylation pathways and temporarily interfered with the energy supply in *M. persicae*. Notably, the number and functional distribution of binding proteins for Cry1Ab1 in *M. persicae* are less extensive than those for Cry41-related [10]. Moreover, only a small set of pathways, primarily involving immune defense and oxidative phosphorylation, showed temporary enrichment 24 h after exposure to the Cry1Ab1 protein in *M. persicae* and no significant pathways were enriched at 48 h. These findings suggested that the Cry1Ab1 protein might have a brief and limited impact on *M. persicae*. The observed results might be explained by either a dispersed impact of Cry1Ab1 on gene expression, rather than a focused biological effect, or a transition of molecular changes to the protein level occurring at 48 h.

Additionally, a recent study revealed the lethal mechanisms of active Cry protein against *M. persicae*. Cry41-related protein exhibited moderate toxicity against *M. persicae* by enhancing Cathepsin B activity and thus accelerating aphid cell apoptosis [10]. The insecticidal mechanism was further validated by examining the effects of Cry41-related mutants (both positive and negative) on Cathepsin B enzyme activity, which correlated with their insecticidal efficacy [47]. It was reported that Cathepsin B could cleave and deactivate antiapoptotic proteins, thereby participating in the caspase-initiated apoptotic pathways [48,49]. In the present study, three *Cathepsin B-like* genes exhibited differential expression and were classified into Cluster 1 of the DEGs. The expression levels of these three genes were significantly up-regulated at 24 and 48 h post-treatment with the Cry1Ab1 protein compared to the control group. However, they were all enriched in the lysosomal and autophagy-animal pathways. Lysosomes and autophagy mainly play a crucial role in the development of organisms and the defense against foreign substances and stress [36,38]. Therefore, the up-regulation of the three *Cathepsin B-like* genes was likely associated with the activation of the immune defense but was not involved in the apoptosis process of aphid cells. This also accounted for the discrepancy with previous findings that a Cathepsin B-like protease identified in *H. armigera* exhibited an insecticidal efficacy when expressed either indiscriminately or at levels significantly higher than those under normal physiological conditions [50]. Therefore, the up-regulation of the three *Cathepsin B-like* genes triggered the defense system rather than accelerated cell apoptosis in aphids, consistent with the insensitivity of *M. persicae* to the Cry1Ab1 protein.

Additionally, the Cry1Ab protein has been reported to have no effects on the reproduction and development of various aphid species. Specifically, the Cry1Ab protein did not impact the reproductive and developmental parameters of the non-target aphid *Sitobion avenae* (Homoptera: Aphididae), such as the intrinsic rate of natural increase (*r*), offspring production, longevity, apterous survivorship, finite rate of increase, and doubling time [18]. Transgenic Bt maize expressing the Cry1Ab protein did not adversely affect the biological parameters of the aphid *Rhopalosiphum maidis*, including the rate of alate production, nymph mortality, nymph development, adult longevity, spawning period, and fecundity [25,35]. The Cry1Ab protein expressed in Bt maize did not significantly influence the population size or dynamics of the bird cherry-oat aphid *Rhopalosiphum padi* [24,51]. There were no adverse effects on the life-table parameters of *Aphis gossypii* after 37 generations of rearing on transgenic Bt cotton that expressed the Cry1Ab/Ac fusion protein [20]. Similar to Cry1Ab1, Cry7Ab4 is non-toxic to *M. persicae* but induces immune gene expression. However, a key difference lies in the involvement of HSP60-mediated protection, which likely contributes to the low sensitivity of *M. persicae* to Cry7Ab4 [52]. In contrast, Cry5Ba2, as recently reported, likely exhibits a distinct mode of action by binding to the apical surface of midgut microvilli and leading to significant reductions in survival and fecundity in *M. persicae* [53]. Additionally, while the Cry1Ab protein has a minimal impact on the growth rates of *Acyrthosiphon pisum*, other Cry proteins such as Cry3A, Cry4, and Cry11A significantly inhibit its growth rates [23].

**Figure 8:**
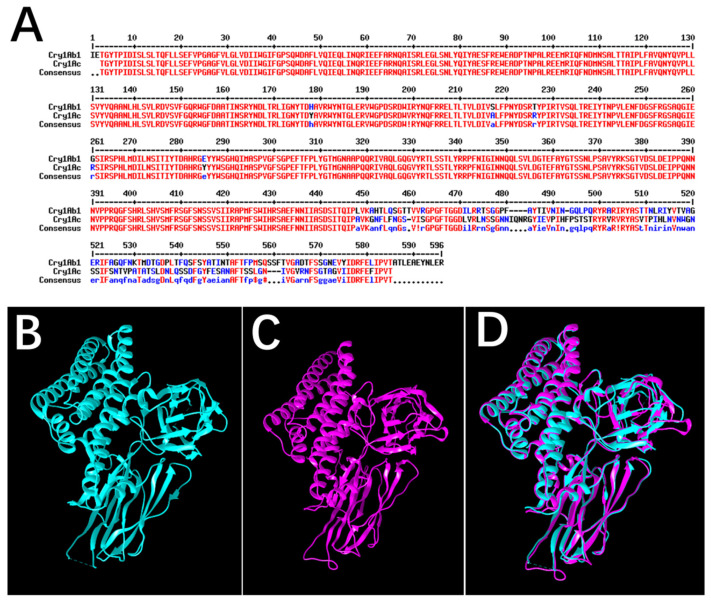
Comparisons of the amino acid sequences and three-dimensional structures of Cry1Ab1 and Cry1Ac proteins. (**A**): Comparisons of the amino acid sequences of Cry1Ab1 and Cry1Ac proteins; (**B**): Three-dimensional model structure of Cry1Ab1 protein (PDB: 6DJ4); (**C**): Three-dimensional model structure of Cry1Ac protein (PDB: 4ARX); (**D**): Comparisons of the three-dimensional structures of Cry1Ab1 and Cry1Ac proteins. Blue bars represent Cry1Ab1, and purple bars represent Cry1Ac. In the comparisons of the amino acid sequences of two proteins, Red, blue, and black represent high consensus, low consensus, and neutral, respectively. In the consensus line, highly conserved residues are depicted in red and denoted by uppercase letters, while weakly conserved residues are indicated in blue and marked with lowercase letters. Neutral residues are represented in black and symbolized by characters such as “#” or “$”. Positions lacking conserved residues are designated by a dot.

Notably, the expression of the Cry1Ab protein in Bt broccoli had no any significant impact on the survival and life-table parameters of *M. persicae*, such as generation time, number of nymphs, and daily fecundity [27]. Paula and Andow [22] showed that the Cry1Ac and Cry1F proteins reduced the net population growth rate of *M. persicae*. Cry1Ab1 and Cry1Ac are both classified as Cry1A proteins, and we conducted a comparison of the amino acid sequences and three-dimensional (3D) structures of these two proteins. The results showed that the amino acid sequence homology between the Cry1Ab1 and Cry1Ac proteins was 84.39% (Figure 8A) through MultAlin (http://multalin.toulouse.inra.fr/multalin/) (accessed on 6 January 2025), suggesting a high sequence similarity between the two proteins. 3D structures of the Cry1Ab1 protein (PDB: 6DJ4) and Cry1Ac protein (PDB: 4ARX) were displayed (Figure 8B,C), and were compared (Figure 8D) using UCSF Chimera. The comparison of 3D structures indicated that the Cry1Ab1 protein and the Cry1Ac protein exhibited certain structural similarities, but remarkable differences in Domain Ⅱ and Domain Ⅲ. In the current Cry protein insecticidal model, Domain II, the central region composed of three antiparallel β-sheets, is crucial for receptor recognition. Domain III, a sandwich structure of two antiparallel β-sheets, is believed to be involved in receptor binding and pore formation [54,55]. Both domains are key in determining insect specificity through specific interactions with insect gut proteins [56]. Saturation mutagenesis studies have confirmed the essential roles of Domain II and Domain III in Cry protein insecticidal activity [57,58,59]. Recent research on Cry non-receptor binding proteins, including the Cry41-related protein in *M. persicae* [46,60], Cry7Ab4 protein in *Plutella xylostella* [61], and Cry1Ab protein in *P. xylostella* and *Spodoptera exigua* [62], has shown that Domain II and Domain III probably participated in the protein binding. Therefore, the differences in Domain II and Domain III between Cry1Ab and Cry1Ac are likely responsible for their distinct impacts on *M. persicae*. Particularly, the significant variation in Domain III may be the primary cause (Figure 8D). Studies have shown that swapping Domain III may result in proteins with different insect target specificities [4]. Thus, we speculated that the structural differences (particularly in Domain III) between Cry1Ab1 and other Cry proteins like Cry1Ac resulted in Cry1Ab1 binding to fewer functional proteins from *M. persicae*. As a consequence, the metabolic processes in *M. persicae* were less affected, leading to no significant impact on the survival and reproductive development of *M. persicae*.

## 4. Materials and Methods

### 4.1. Experimental Materials

*M. persicae* were reared on *Brassica chinensis* L., which was cultured in an artificial climate chamber (JINGHONG, Shanghai, China) with a temperature of 20 ± 2 °C, relative humidity of 70–80%, and a photoperiod of L:D = 16:8. The *Escherichia coli* strain expressing the Cry1Ab1 protein was preserved in our lab. The purification of recombinant proteins was carried out using ProteinIso Ni-NTA resin (TranGen, Beijing, China).

### 4.2. Preparation of the Cry1Ab1 Protein

The expression and purification of the Cry1Ab1 protein were performed using the methods described [62]. Briefly, Recombinant pEASY-Cry1Ab1 positive clones were confirmed by colony PCR and grown in Luria–Bertani (LB) medium (100 mg/L ampicillin) at 37 °C. Protein expression was induced by 0.1 mM Isopropyl β-D-1-Thiogalactopyranoside (IPTG) at an OD_600_ of 0.6. After 20 h of incubation at 20 °C, cells were harvested and disrupted by sonication, and inclusion bodies were isolated. The proteins were purified using ProteinIso Ni-NTA resin (TransGen, Beijing, China), with refolding conducted through urea gradient dialysis following the published protocol [63].

### 4.3. Bioassay of the Cry1Ab1 Protein Against M. persicae

The toxicity of Cry1Ab1 protein to *M. persicae* was evaluated using the leaf-dip method [64]. Cry1Ab1 protein was diluted to concentrations of 10, 100, 150, and 250 mg/L with distilled water, and a Cry1Ab1 protein-free solution was used as the negative control. *Brassica chinensis* L. 6-week-old leaves measuring approximately 5.5 cm × 4 cm (length × width), were dipped into the solutions for 15 s and then dried in a cool, ventilated area. The treated leaves were placed in dishes (9 cm diameter, 2 cm height) containing 1.8% agar, with the underside facing up (one leaf per dish). 30 fourth instar aphid nymphs were then individually transferred into each dish. Each treatment had three replicates. Mortality was recorded at 24 and 48 h.

Additionally, the toxicity of the Cry1Ab1 protein to *M. persicae* was also assessed using the membrane capsule method [47]. A stretched 8 × 8 cm^2^ parafilm was secured over one end of a cylindrical container (4 cm diameter, 3 cm height, and 2 mm wall thickness). A 2 mL diet containing 10, 100, 150, or 250 mg/L Cry1Ab1 protein was added and covered with another parafilm layer to form a capsule, with a Cry1Ab1 protein-free diet being as negative control. 30 fourth instar aphid nymphs were placed in the lower part of each capsule, with each treatment replicated three times. The other end of the cylinder was sealed with a 6 × 6 cm^2^ parafilm perforated with 20–30 ventilation holes. Mortality was recorded every 24 h over a total period of 168 h (7 days). All of the aphids for the experiments were kept at 18–20 °C, 80% humidity, and a 16:8 light:dark cycle. Aphids were gently probed daily to assess mortality (non-responders were considered dead), and data were recorded for analysis.

In this bioassay, the two methods—the leaf dipping method and the membrane capsule method—were employed to evaluate the toxicity of the Cry1Ab1 protein to *M. persicae*. The leaf dipping method was chosen to simulate natural feeding conditions, ensuring ecological relevance, while the membrane capsule method provided controlled and quantifiable exposure for mechanistic studies. The leaf dipping method allows uniform protein distribution on leaf surfaces but may introduce variability in protein absorption. In contrast, the membrane capsule method ensures precise dosage delivery but lacks ecological relevance. Combining these methods balances natural exposure scenarios with experimental precision, offering a comprehensive assessment of Cry1Ab1 protein toxicity.

### 4.4. Pull-Down Experiment and LC-MS/MS Analysis

The *M. persicae* homogenate was extracted from 200 healthy fourth instar *M. persicae* nymphs using a tissue grinder. The pull-down experiment was performed using the method described [62], and the results of the separation were analyzed by sodium dodecyl sulfate-polyacrylamide gel electrophoresis (SDS-PAGE), followed by silver staining. The bands that appeared in all treatment groups, but not in the control group, were excised and sent to the Beijing Huada Protein Research and Development Center (HPRC) for LC-MS/MS analysis. HPRC performed a search of the LC-MS/MS data against the uni-Aphidoidea 33,385 database (52,503 sequences and 17,662,111 residues) using the MASCOT search engine (Matrix Science, London, UK). The pull-down and LC-MS/MS experiments were conducted in triplicate to ensure reproducibility. The protein identification and quantification were performed using MaxQuant software (version 1.5.2.6). Statistical significance was determined using a two-tailed Student’s *t*-test (*p* < 0.05), with multiple testing correction applied via the Benjamini-Hochberg method (FDR < 0.05). The results were submitted to the BLAST program of the National Center for Biotechnology Information (NCBI) (https://blast.ncbi.nlm.nih.gov/Blast.cgi) (accessed on 10 September 2022), and the accession numbers and related information of the binding proteins were subsequently obtained.

### 4.5. Functional Analysis of Cry1Ab1-Binding Proteins

The STRING database integrates protein-protein interaction data from numerous predicted and known organisms, including direct (physical) interactions with specific biological functions and indirect (functional) interactions [65]. Therefore, the sequences of those proteins binding to the Cry1Ab1 were submitted to the STRING server for their functional analysis.

### 4.6. RNA Sequencing

To investigate the impact of Cry1Ab1 on the transcriptome of *M. persicae*, fourth instar nymphs were exposed to 50 μg/mL Cry1Ab1 protein for 24 and 48 h (30 aphids per group were dissected). Each experimental condition included three independent biological replicates. Total RNA was isolated from the treated nymphs using the TRIZOL reagent (Invitrogen, Carlsbad, CA, USA) according to the manufacturer’s protocol. RNA concentration was determined using a NanoDrop 2000 C spectrophotometer (Thermo, Waltham, MA, USA), and RNA integrity was assessed through 1.2% agarose gel electrophoresis. High-quality RNA samples were then employed for cDNA synthesis and Illumina library preparation, performed by Biomarker Technology Co., Ltd. (Beijing, China).

### 4.7. Transcriptome Assembly and Functional Annotation

The raw data underwent quality control using SeqPrep software (https://github.com/jstjohn/seqprep) (accessed on 10 August 2024) to generate high-quality clean reads. These clean reads were then assembled into transcripts and unigenes using Trinity software (version v2.8.5 https://gith ub.com/trinityrnaseq/trinityrnaseq) (accessed on 10 August 2024). Initially, Trinity deconstructed sequencing reads into short fragments known as k-mers, which were then extended into longer contigs by overlapping these fragments to form a comprehensive set. Subsequently, de Bruijn graphs were utilized to reconstruct full-length transcript sequences from these contig sets. For functional annotation, Diamond software (version 0.9.24 https://github.com/bbuchfink/diamond) (accessed on 12 August 2024) was employed to align the unigene sequences against multiple databases, including the Non-Redundant Protein Sequence Database (NR), Swiss-Prot Protein Sequence Database (Swiss-Prot), Cluster of Orthologous Groups of Proteins (COG), and the Kyoto Encyclopedia of Genes and Genomes (KEGG). The KEGG Orthology Based Annotation System (KOBAS) was used to map unigenes to KEGG orthologs. For Gene Ontology (GO) classification, InterProScan was applied to interpret results using the integrated InterPro database. The amino acid sequences of unigenes were predicted, and HMMER software (version 3.2.1 http://hmmer.org/) (accessed on 20 August 2024) was used to compare them against the Pfam database to annotate the unigenes.

### 4.8. DEGs Analysis

The identified genes were analyzed using DESeq software (version 1.10.1 http://bioconductor.org/packages/stats/bioc/DESeq2/) (accessed on 24 August 2024), with a threshold of *p* < 0.05 and an absolute |log2 (fold change)| ≥ 1 to determine DEGs. Functional classification of these DEGs was carried out using the GO database, while GO enrichment analysis was performed with GOATOOLS. GO functions with a *p*-value ≤ 0.05 were considered significantly enriched. Additionally, the KEGG database was employed for both functional classification and enrichment analysis of DEGs, with pathways showing a *p*-value ≤ 0.05 deemed to be significantly enriched.

### 4.9. Statistical Analysis

Statistical analyses were conducted using SPSS Statistics 16.0 software. One-way ANOVA was used to assess the significance of the data. The LSD method was utilized to determine significant differences between groups at significance levels of *p* < 0.05. GraphPad Prism 8.0 software was used to make graphs.

## 5. Conclusions

Overall, we conducted multiple studies, including the molecular mechanism stem from Cry-binding proteins function and transcriptomic analysis as well as structural analysis, to expose the underlying causes for the ineffectiveness of the Cry1Ab1 protein on *M. persicae*. This study provided initial clues about the differential effects of Cry proteins on non-target aphids. Future research should further explore Cry1Ab1′s effects on non-target organisms, its molecular mechanisms, and field efficacy. Cry1Ab1 could be integrated into IPM strategies or engineered into crops for sustainable pest control. This study will contribute to enriching the safety assessment framework for Bt in non-target organisms.

## Figures and Tables

**Table 1:** LC-MS/MS identification for Cry1Ab1-binding proteins in *M. persicae*.

Sample	Accession Number	Score	Protein Description
1	tr|T1UMS9|T1UMS9_APHGO	82	Beta-actin OS = *Aphis gossypii*
2	tr|J9K1R5|J9K1R5_ACYPI	31	GPN-loop GTPase 2 OS = *Acyrthosiphon pisum*
3	tr|J9M0Y0|J9M0Y0_ACYPI	29	Uncharacterized protein OS = *Acyrthosiphon pisum*
4	tr|Q5XUA1|Q5XUA1_TOXCI	28	ATP synthase subunit alpha OS = *Toxoptera citricida*

## Data Availability

Data are contained within the article and Supplementary Materials.

## Supplementary Materials

The following supporting information can be downloaded at: https://www.mdpi.com/article/10.3390/ijms26072924/s1.
