# Supplementary material for: Potential Mechanisms Underlying the Minimal Impact of Cry1Ab1 Protein on Myzus persicae
Source: Int J Mol Sci. 2025 Mar 24;26(7):2924. doi: 10.3390/ijms26072924 (PMC11988580; doi:10.3390/ijms26072924)
Supplement: Supplementary file 1 [file ijms-26-02924-s001.zip › ijms-3497565-supplementary.pdf]

Supplementary information

Potential Mechanisms Underlying the Minimal Impact of Cry1Ab1 Protein on *Myzus persicae*

Liang Jin[1, 2], Binwu Zhang[2], Luis Carlos Ramos Aguila[3], Jingwen Lu[2], Xueke Gao[1], Junyu Luo[1], Jinjie Cui[1, *], and Yi Lin[2, *]

[1] Research Base of Zhengzhou University, State Key Laboratory of Cotton Bio-breeding and Integrated Utilization, Institute of Cotton Research, Chinese Academy of Agricultural Sciences, Anyang 455000, China

[2] Fujian Provincial Key Laboratory of Biochemical Technology, Department of Bioengineering & Biotechnology, College of Chemical Engineering, Huaqiao University, Xiamen 361021, China

[3] State Key Laboratory of Ecological Pest Control for Fujian and Taiwan Crops, College of Plant Protection, Fujian Agriculture and Forestry University, Fuzhou 350002, China;

*Correspondence: cuijinjie@caas.cn (J. C.) and lyhxm@hqu.edu.cn (Y. L.).

Table:

Table S1. Statistical summary of transcriptome sequencing data and the outcomes of the assembly.

Sample	Raw reads	Error rate(%)	GC content (%)	Q30 (%)	Clean reads	Mapped reads	Mapped ratio(%)
CK 24h 1	44100732	0.0118	45.51	96.36	43749174	41134654	94.02
CK 24h 2	43575014	0.0117	42.96	96.57	43272324	40776045	94.23
CK 24h 3	43301634	0.0117	44.43	96.54	42971704	40488358	94.22
Cry1Ab1 24h 1	43334118	0.0118	43.94	96.45	43004248	40492648	94.16
Cry1Ab1 24h 2	42616394	0.0119	46.56	96.27	42283270	39619742	93.7
Cry1Ab1 24h 3	44139822	0.012	47.19	96.06	43795004	40598584	92.7
CK 48h 1	42715730	0.0119	46.09	96.16	42389240	39784481	93.86
CK 48h 2	43434914	0.0121	46.91	95.91	43067548	40182572	93.3
CK 48h 3	44525850	0.0117	43.44	96.54	44233960	41576962	93.99
Cry1Ab1 48h 1	44712654	0.0116	40.66	96.68	44440380	42058336	94.64
Cry1Ab1 48h 2	44592884	0.0118	44.64	96.47	44289958	40439395	91.31
Cry1Ab1 48h 3	44662718	0.0117	42.38	96.53	44314980	41700447	94.1

Figures:

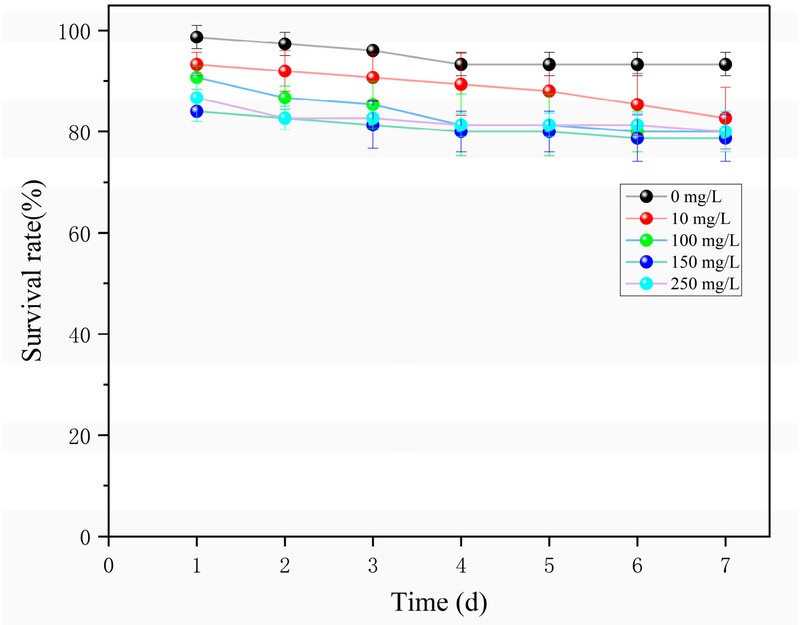

Figure S1. Toxicity of Cry1Ab1 against *M. persicae* using membrane capsule method. Fourth instar *M. persicae* nymphs were fed with artificial diets mixed with Cry1Ab1 protein at concentrations of 0, 10, 100, 150, and 250 mg/L, respectively. The survival was continuously observed for 7 days, with daily records of survival being maintained.

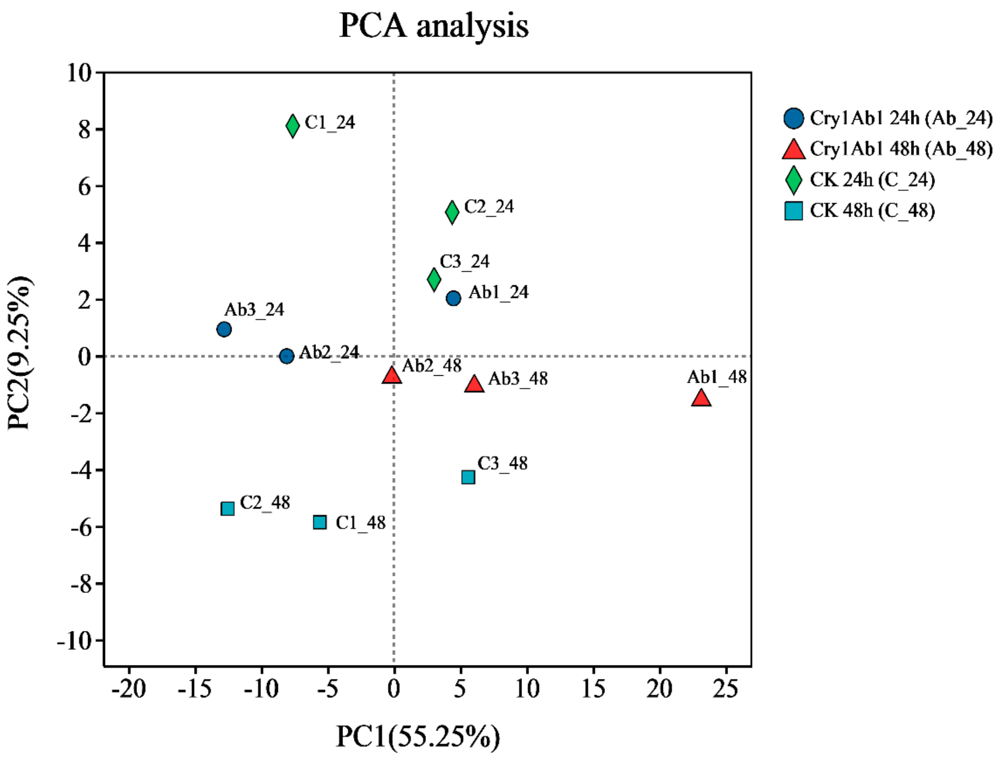

Figure S2. Principal coordinates analysis (PCA) ordination.
